# Exploring the potential anti-apoptotic effects of traditional Chinese medicine in intervertebral disc degeneration: mechanisms and therapeutic prospects

**DOI:** 10.3389/fphys.2025.1617215

**Published:** 2025-08-04

**Authors:** Kuaixiang Zhang, Lei Wan, Malik Ihsan Ullah Khan, Feifei Pu, Man Liu, Zhiqiang Zhao, Jitian Li

**Affiliations:** ^1^ The Third Affiliated Hospital of Xinxiang Medical University, Clinical Medical Center of Tissue Engineering and Regeneration, Zhongyuan Regenerative Medicine Laboratory, Xinxiang Medical University, Xinxiang, China; ^2^ Henan Luoyang Orthopedic Hospital (Henan Provincial Orthopedic Hospital), Henan University of Chinese Medicine, Zhengzhou, China; ^3^ The Second Affiliated Hospital of Luohe Medical College, Luohe, China; ^4^ Henan International Joint Laboratory of Prevention and Treatment of Degenerative Spinal Diseases with Traditional Chinese Medicine, Zhengzhou, China; ^5^ Department of Orthopedic, Wuhan No. 1 Hospital, Wuhan, China; ^6^ Luoyang Orthopedic Hospital Affiliated to Hunan University of Chinese Medicine, Changsha, China

**Keywords:** intervertebral disc degeneration, apoptosis, death receptor pathway, mitochondrial pathway, endoplasmic reticulum stress pathway

## Abstract

Intervertebral disc degeneration (IDD) stands as one of the primary culprits behind low back pain and disability, imposing substantial burdens on individual health, families, and societal wellbeing. The multifactorial etiology and complex pathology of IDD pose significant challenges, with molecular mechanisms still not fully elucidated. A key aspect of the pathogenesis of IDD involves programmed cell death, specifically apoptosis, which exacerbates the condition by fostering the apoptosis of intervertebral disc (IVD) cells and accelerating the degradation of the extracellular matrix (ECM). Conversely, inhibiting apoptosis signalling pathways has emerged as a promising therapeutic strategy for IDD. Recent research has highlighted the potential of traditional Chinese medicine (TCM) to alleviate IDD at the genetic level by modulating apoptotic pathways. This review integrates the intricate mechanisms of IDD-induced cell apoptosis, the relevant targets implicated in IVD cell apoptosis, and the latest advancements in TCM-based treatments, drawing from a comprehensive analysis of literature sourced from the PubMed, China National Knowledge Infrastructure (CNKI), and Web of Science databases. This study aims to offer fresh perspectives and innovative concepts for pharmacological interventions and to serve as a valuable reference for ongoing and future research endeavors. As the field of antiapoptotic research progresses, there is increasing urgency to identify safe, effective, and economically viable compounds from the vast array of natural resources, including plants and animals, to support the prevention and management of IDD. This approach not only aligns with the principles of sustainable development but also holds the promise of enriching the therapeutic armamentarium against IDD.

## 1 Introduction

Intervertebral disc degeneration (IDD) stands as the foremost contributor to chronic low back pain (LBP), a condition that significantly diminishes quality of life and can lead to disability. It is estimated that approximately 80% of the population will experience LBP at some point in their life (Zehra et al., 2022; Li et al., 2021; Cabral et al., 2022). To a great degree, IDD results in pathological and anatomical structural changes in the spine, which increases the risk of LBP.

However, current clinical treatments, including manual therapy, pharmacological, and surgical interventions, often fail to provide a fundamental resolution to IDD and carry the risk of recurrence (Zhu et al., 2020; Chu and Sabourdy, 2023). Most medications available today address only symptoms, failing to treat the underlying causes of IDD or significantly slowing its progression. As the condition advances, if conservative treatments prove ineffective, patients are frequently left with surgery as their only option. Surgical procedures, while sometimes necessary, are invasive, expensive, and pose the risk of exacerbating damage to other areas of the spine. Consequently, there is a pressing need for innovative therapeutic approaches that address the etiology and pathogenesis of IDD, potentially transforming the current treatment paradigm.

The occurrence of IDD is strongly related to increasing age, but trauma, lifestyle factors, and certain genetic factors can increase the likelihood of IDD progression (Krut et al., 2021). Upon the onset of IDD, the intervertebral disc (IVD) undergoes significant morphological alterations, characterized by decreased proteoglycan levels, cell death within the nucleus pulposus (NPC), reduced water content in the nucleus pulposus (NP), and degradation of the extracellular matrix (ECM). While the exact etiology of IDD remains incompletely understood, its hallmark pathological features include extracellular matrix (ECM) degradation, apoptosis, and cellular senescence (Cazzanelli and Wuertz-Kozak, 2020). Consequently, inhibiting the apoptotic pathway within the IVD represents a promising strategy for the treatment of IDD, offering a potential avenue to halt or even reverse the degenerative process.

In traditional Chinese medicine (TCM) theory, IDD is categorized under the broader term “TCM arthralgia syndrome”. Despite the absence of a specific disease name for IDD in TCM, the use of TCM in the prevention and treatment of related conditions has a rich history spanning over a thousand years (Zhu et al., 2020). Within this framework, IDD pathogenesis is principally attributed to core imbalances such as Kidney Essence Deficiency (Shen Jing Kui Xu), Qi and Blood Stagnation (Qi Zhi Xue Yu), and Pathogenic Dampness (Shi Xie). TCM posits that the Kidneys govern bone and marrow; their deficiency weakens the spine’s foundation. Stagnation of Qi and Blood impedes nourishment and circulation to the IVDs, while Dampness accumulation obstructs meridians and promotes degeneration. This conceptualization directly links the TCM understanding of systemic dysfunction to the pathological changes observed in IDD. Thus, TCM interventions target root causes (Ben) via kidney tonification, Qi/blood activation, and dampness resolution—beyond symptomatic relief (Biao). Despite this theoretical foundation, however, no systematic review has critically analyzed the antiapoptotic mechanisms of TCM in modulating these pathological processes.To address this gap, we conducted a comprehensive literature review exploring TCM-mediated apoptosis inhibition in IDD. PubMed, China National Knowledge Infrastructure (CNKI) and Web of Science were searched for animal studies using “apoptosis” and “IDD” to elucidate molecular targets driving IDD pathology, accelerate evidence-based drug discovery and inform design criteria for clinical trials.

## 2 Classical apoptosis pathway

**FIGURE 1:**
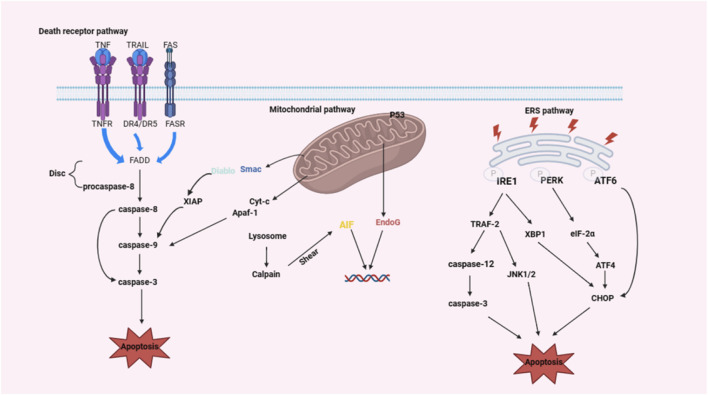
Mechanism of the classical apoptotic pathway during disc degeneration (created with BioRender.com). IDD involves three classical apoptotic signaling axes: death receptor-mediated extrinsic pathway, mitochondrial-driven intrinsic pathway, and ERS-activated cascade.

Apoptosis, a highly regulated form of programmed cell death, plays a critical role in maintaining cellular homeostasis (Kiraz et al., 2016). Unlike necrosis-an uncontrolled passive process often associated with pathological damage-apoptosis is a tightly regulated form of programmed cell death (PCD). This active mechanism maintains tissue homeostasis and occurs under both physiological and pathological conditions (D’Arcy, 2019). Physiologically, moderate apoptosis of NPCs may contribute to tissue homeostasis through programmed cell turnover mechanisms, thereby preserving the viscoelastic properties and biomechanical functionality of IVD. However, during degenerative disc disease progression, excessive apoptotic cascade activation disrupts extracellular matrix architecture via accelerated proteoglycan depletion and collagen network disorganization, ultimately manifesting as disc height reduction, mechanical instability, and nociceptive signaling amplification through pro-inflammatory cytokine release (e.g., IL-1β, TNF-α) and nerve ingrowth potentiation. Apoptosis can be initiated either by external signals or by internal triggers. External signals, such as those from the immune system or other cells, can activate death receptors on the cell surface, whereas internal signals can arise from cellular stress or DNA damage. Disruption of the apoptotic process can lead to various diseases, including degenerative conditions in humans (Zhao et al., 2006). In the context of intervertebral disc degeneration (IDD), three primary pathways are involved in apoptosis: the exogenous death receptor pathway, the endogenous mitochondrial pathway, and the endoplasmic reticulum stress (ERS) pathway (Tschoeke et al., 2008; Chen et al., 2019). These pathways contribute to IDD by mediating the apoptosis of IVD cells (Figure 1).

### 2.1 Death receptor pathway

The Nomenclature Committee on Cell Death (NCCD) defines extrinsic apoptosis as a type of regulated cell death (RCD) stimulated by perturbations of the extracellular microenvironment sensed by plasma membrane receptors, propagated by caspase-8 and precipitated by executioner caspases, mainly caspase-3 (Galluzzi et al., 2018). This process involves three main families of proteins, including tumor necrosis factor receptor 1 (TNFR1), Fas, and tumor necrosis factor (TNF)-related apoptosis-inducing ligand (TRAIL) (Herold and Strasser, 2014). The interaction between these death receptors and their respective ligands, such as TNF-α and FasL, initiates a cascade of signalling events that activate the caspase protease family, which plays a crucial role in the apoptotic process within cells. Once activated, caspase proteases cleave specific substrates, culminating in programmed death of the cell.

In addition, the death receptor pathway can also induce the expression of prosurvival genes through the activation of certain signalling molecules, thereby allowing the cell to survive. However, the primary mechanism of this pathway is the induction of apoptosis, which is characterized by the activation of “initiating” caspase-8 and “executing” caspase-3 (Tummers and Green, 2017; Thorburn, 2004). When the death receptor is activated by its corresponding ligand, it signals through specific adaptor proteins, such as Fas-associated death domain protein (FADD), which in turn activates caspase-8 through facilitating the oligomerization of procaspase-8. Fas-FasL, FADD and procaspase-8 form a death-inducing signalling complex (DISC) (Mahdizadeh et al., 2021). Subsequently, caspase-8 can directly or indirectly activate caspase-3, which is the main executor of apoptosis and can cut a variety of important cellular proteins, leading to the destruction of cell structure and function, and eventually triggering PCD-induced apoptosis.

#### 2.1.1 Fas/FasL pathway

As reported by Sun et al. (2013), the Fas mRNA level was found to be significantly elevated in degenerated NPCs compared with normal cells. These findings suggest that the Fas/FasL signalling pathway may play an important role in the pathogenesis of IDD. In addition to promoting apoptosis in NPCs, the death receptor pathway has also been shown to induce apoptosis in annulus fibrosus (AF) cells (Chen et al., 2015) and cartilaginous endplate (CEP) cells (Wang et al., 2011), contributing to the development of IDD. Research on the role of the death receptor pathway in IDD has focused mainly on the Fas/FasL pathway, which is the focus of this study.

Fas antigen, also known as apo-antigen (CD95), is a type I transmembrane protein belonging to the TNF and nerve growth receptor protein superfamily (Sharma et al., 2000). Fas consists of a cytoplasmic C-terminal domain, an extracellular N-terminal portion, and a transmembrane region. A key component of apoptotic signalling is the death domain (DD), which is located in the intracellular region (Kaufmann and Earnshaw, 2000). FasL is the ligand of Fas, which is a type Ⅱ transmembrane protein belonging to the TNF family (Griffith et al., 1995). FasL is composed of three parts: the extracellular region, the transmembrane region and the intracellular area. The intracellular portion of FasL, is referred to as the DD, which is homologous to Fas, whose intracellular region can bind to a protein known as FADD. When death receptors bind to specific death ligands, they receive extracellular death signals and activate intracellular apoptotic mechanisms. Fas recruits the adaptor protein FADD in the cytoplasm through the interaction between the DD of its intracellular segment and the DD at the carboxyl terminus of FADD. FADD interacts with caspase-8 through its death effector domain (DED), facilitating the formation of the DISC. This interaction activates caspase-8, which subsequently triggers the activation of downstream caspase family members, culminating in the execution of apoptosis (Siegel et al., 2000).

Buyang Huanwu decoction has been shown to slow the progression of IDD by blocking the death receptor pathway and inhibiting the overexpression of the Fas/FasL gene, thereby promoting the regeneration of vascular buds and increasing the proteoglycan content in the CEP (Lv and Wu, 2011; Lv and Wu, 2012). The Yiqi Huayu recipe was demonstrated to decelerate cell apoptosis by downregulating Bax and caspase-8 while upregulating Bcl-2 expression (Zhou et al., 2005). In rat endplate chondrocytes subjected to anti-Fas antibody treatment, this formula alleviated apoptosis, suggesting potential modulation of the Fas/FasL pathway—a mechanism requiring further experimental validation. Duhuo Jisheng decoction reduces apoptosis by inhibiting the activation of Fas-mediated apoptosis pathways Ⅰ and Ⅱ, decreasing the expression levels of Fas, FasL, Bax, Bid, caspase-3 and caspase-8 and increasing the expression level of B-cell lymphoma-2(Bcl-2) (Jianhua et al., 2016). The mechanism by which Yaotu granules alleviate IDD may involve reducing the expression of the Fas/FasL gene in the NP in a dose-dependent manner in a rabbit lumbar IDD model (Shenghua et al., 2017). Paeoniflorin, a pinane monoterpene bitter compound found in Radix Paeoniae Alba, Radix Paeoniae Rubra, and Radix Paeoniae Suffruticosa, decreases the gene expression of Fas, caspase-3, and caspase-8 in annulus fibrosus cells, effectively inhibiting the Fas/FasL signalling pathway (Shaoqing et al., 2012).

Fas plays an important role in the production of proinflammatory cytokines in IVD cells (Yamamoto et al., 2013). In turn, inflammatory mediators such as reactive oxygen species (ROS), nitric oxide (NO), interleukin (IL)-1β, tumor necrosis factor-α (TNF-α), and Fas are closely related to chondrocyte apoptosis (Xiao et al., 2023). Moreover, inflammatory factors such as TNF-α, IL-1, IL-2 and lipopolysaccharide (LPS) are essential factors leading to IDD (Molinos et al., 2015). Multiple studies have demonstrated that IL-1β and TNF-α are essential factors in the apoptosis of IVD cells, which was confirmed by Wang et al. (2020). However, the precise mechanism of the interaction between the Fas/FasL signalling pathway and inflammatory mediators remains to be elucidated. Mounting evidence suggests that inflammatory factors associated with IDD and the Fas receptor may exert synergistic effects. Inflammatory factor inhibition may partially inhibit the Fas signalling pathway in IVDs. Chinese herbal medicines, including Bushen Huoxue Decoction (Shuaihua et al., 2015; Shuaihua et al., 2018; Ren et al., 2017), Liuwei Dihuang Pills (Guohua et al., 2015), Shentong Zhuyu Decoction (Demin et al., 2019), Shujin Qubi Decoction (Yuxiang et al., 2018), Tongluo Decoction (subred et al., 2015), Zhiqiao Gancao Decoction (Jiangtao et al., 2016), and Fufangqishe-Pill (Liang et al., 2010), have been shown to alleviate IDD by reducing the expression of IL-1β and (or) TNF-α *in vitro* experiments and animal models. In addition, the monomeric components extracted from TCMs also have the same effect. For example, naringin is a flavonoid compound isolated from tomatoes, grapefruit, and related citrus fruits that inhibits the overexpression of inflammatory cytokines (Devraj et al., 2019).

The IVD is the largest avascular organ in the human body. It consists of three parts: the NP, the AF, and the CEP. The central NP has been surrounded by AF since its formation and is sandwiched between the two CEPs. This unique structure isolates the NP from the host immune system. In addition, molecular factors expressed in IVDs have been shown to exert inhibitory effects on immune cell and cytokine infiltration. Therefore, the IVD has been identified as an immunologically privileged organ (Sun et al., 2020). Studies have shown that the Fas/FasL apoptotic mechanism plays an important role in maintaining the homeostasis of the normal immune response and that dysregulation of the Fas/FasL apoptotic pathway can lead to some diseases (Lynch et al., 1995). Accumulating evidence shows that FasL may play an important role in maintaining IVD immune privilege by inducing immune cell apoptosis (Lynch et al., 1995). Although the main mechanism of IDD-related immunity and Fas-mediated apoptosis has not been fully elucidated, TCM provides a new target for the treatment of IDD through immune privilege mechanisms. Radix Astragali, which is isolated from the dried root of Astragalus membranaceus, is considered to be an immunomodulator that can enhance the function of the human immune system. In clinical applications, the reuse of astragalus has a significant effect on the treatment of nerve paralysis symptoms in the lower limbs, such as acid anaesthesia after lumbar spinal stenosis (Jiang et al., 2011).

In conclusion, Fas pathway inhibition may be an effective way to treat IDD. Therefore, it is necessary to further investigate the mechanism of action of TCM in relieving IDD through the death receptor pathway.

#### 2.1.2 TRAIL/TRAILR pathway

Death receptors 4 (DR4) and 5 (DR5) are two TRAIL receptors known for their ability to transduce apoptotic signals and initiate apoptosis upon binding to TRAIL. Emerging evidence suggests that aberrant activation of the TRAIL/TRAILR pathway contributes to IDD, with elevated expression levels of TRAIL and its receptors observed in degenerated NPCs. This highlights a promising yet underexplored avenue for future research aimed at developing novel TCM-based therapeutics that specifically target the TRAIL/TRAILR axis. However, despite growing insights in understanding this pathway, there remains a paucity of herbal interventions that target DR4/DR5 to treat IDD and reverse apoptosis in recent years.

#### 2.1.3 TNF/TNFR pathway

The TNFR superfamily comprises more than 20 type Ⅰ transmembrane proteins with conserved N-terminal cysteine-rich domains (CRDs) in the extracellular ligand-binding region that are specifically activated by the corresponding TNF-like ligand superfamily. One of the remarkable features of the TNFR superfamily is the ability of these receptors to induce apoptosis (Chung et al., 2007). The TNF/TNFR pathway represents one of the most well-characterized apoptotic signalling axes in the pathogenesis of IDD. In degenerative IVD tissues, elevated levels of TNF-α have been consistently associated with increased apoptosis of disc cells, underscoring its pivotal role in disease progression and highlighting TNFR1 as a promising therapeutic target. Despite this mechanistic understanding, there are no relevant studies on the application of TCM to inhibit this pathway to alleviate IDD. Future investigations should focus on identifying novel TCM-derived molecules capable of selectively modulating TNFR1 signaling, potentially offering dual benefits in both apoptosis inhibition and inflammatory regulation in IDD.

### 2.2 Mitochondrial pathway

Endogenous mitochondrial apoptosis is a type of RCD caused by a variety of cytokine microenvironmental disturbances, including (but not limited to) growth factor regression, DNA damage, endoplasmic reticulum (ER) damage, overloading, replication stress, and microtubule alterations or defects in mitosis. The NCCD proposes defining extrinsic apoptosis as a type of planned cell death that is initiated by external or intracellular stimulation and then produced by caspases (primarily caspase-3) as executor caspases (Galluzzi et al., 2018).

Bcl-2 family proteins and caspase family proteins play crucial roles in the regulation of apoptosis, acting as upstream and downstream regulators of mitochondrial function, respectively. Bcl-2 family proteins regulate the permeability of the mitochondrial outer membrane through the synergistic action of proapoptotic and antiapoptotic members, thus triggering the internal pathway of mitochondria (Estaquier et al., 2012). When the mitochondrial membrane potential decreases, cell membrane permeability increases, and proapoptotic factors are released into the cytoplasm. After cytochrome c (Cytc) is released into cells, apoptotic cell complexes are formed with the assistance of ATP and dATP. The apoptotic complex recruits and activates procaspase-9 to form the caspase-9 holoenzyme. Caspase-9 further activates the effector proteins caspase-3 and caspase-7, initiating the caspase cascade that ultimately leads to apoptosis (Oberst et al., 2008; Zacharioudakis et al., 2022). Apoptotic death induced by a second mitochondria-derived activator of caspases (SMAC/DIABLO) and HTRA2/OMI are released from mitochondria and bind to inhibitors of apoptosis proteins (IAPs) (which can inhibit caspase-3 and caspase-7 activation), thereby inhibiting the action of IAPs and indirectly promoting apoptosis (Zacharioudakis et al., 2022).

This pathway involves p53, AIF, Cytc, Smac/DIABLO, the Bcl-2 family, which includes Bcl-2/Bax, PUMA, Bim and Bcl-xl, the IAP family and other proteins that are intimately connected to the mitochondrial apoptosis pathway. In addition, mitophagy and oxidative stress are involved in this process. P53 protein can directly induce mitochondrial outer membrane permeability, leading to Cytc release, by forming complexes with the protective proteins BclXL and Bcl-2 that lead to apoptosis (Mihara et al., 2003). Oxidative stress promotes mitophagy via JNK signalling in the early stages but reduces mitophagy and enhances apoptosis in the later stages (Fan et al., 2019). Mitophagy protects cells from apoptosis by removing abnormal or damaged mitochondria, controlling the quality and quantity of mitochondria, maintaining the dynamic balance of mitochondria, and resisting oxidative stress (Wang et al., 2023a).

The mitochondrial apoptosis pathway presents a promising therapeutic target for a variety of diseases (Gibson and Davids, 2015). Studies focusing on this pathway offer valuable insights that could be applied to the treatment of IDD. By inhibiting the expression of Bax, cleaved caspase-3, caspase-3, and Cyt-c, and increasing the expression of antiapoptotic proteins such as Bcl-2, Bushen Huoxue Formula might alleviate IDD and inhibit the development of inflammatory factors (Gao et al., 2024). Through the protection of IDD against decreased Cytc, Bax, and caspase-3 levels and increased expression of Bcl-2, Bushen Zhuangdu decoction can alleviate IDD via the mitochondrial-mediated apoptosis signalling pathway (Civilization et al., 2019). By suppressing the NLRP3 inflammatory vesicle signalling system, honokiol which is a component extracted from Magnolias officinalis has been shown to benefit the IVD (Tang et al., 2018). Pyrroloquinoline quinone can protect rat NPCs against oxidative stress via a mitochondria-mediated pathway by increasing the expression of Bcl-2, inhibiting the release of mitochondrial Cytc, and decreasing the expression of Bax and cleaved caspase-3 (Yang et al., 2015). Lupeol (Guo et al., 2018a), myricetin (Chen et al., 2023), and Salvia miltiorrhiza (Qin et al., 2020) can alleviate IDD by inhibiting oxidative stress. In addition, naringin (Zhang et al., 2018a), sesamin (Zhang et al., 2024), sinomenine (Gao et al., 2019), and β-ecdysterone (Wen et al., 2019)can alleviate IDD by promoting autophagy in NPCs.

### 2.3 ERS pathway

In addition, the ER is the hub connecting environmental signals and cell biological functions, and ER dysfunction can lead to a variety of diseases. NPC apoptosis is closely related to ERS. The ER is a central organelle located in the cytoplasm and consists of membranes that synthesize, fold, and modify secreted and transmembrane proteins (Chen and Cubillos-Ruiz, 2021). The protein-folding capacity of the ER can be disrupted by intracellular or external factors, leading to increased internal stress and protein misfolding, further leading to ERS. ERS reduces protein synthesis, increases protein folding, and maintains intracellular Ca^2+^ homeostasis, but excessive stress can trigger and promote apoptosis (Oakes and Papa, 2015). By inositol-requiring enzyme 1α (IRE1α), and PRKR-like ER kinase (PERK) activation and activating transcription factor 6 (ATF6), cells are able to detect ER mic reticulum stress and carry out a series of corrective responses that help maintain cell homeostasis (Iurlaro et al., 2016; Ren et al., 2021).

IRE1 is a protein distributed on the ER membrane. As the unfolded protein accumulates in the ER, the cytoplasmic domain of IRE1 is phosphorylated, which activates TRAF2, which subsequently phosphorylates and activates ASK1, activates JNK, and triggers apoptosis (Chen and Cubillos-Ruiz, 2021). On the other hand, activated TRAF-2 causes caspase-12 activation and initiates the caspase cascade, which in turn causes apoptosis. In the IRE1α-X box binding protein 1 (XBP1) signalling pathway, cleavage of XBP1 mRNA by IRE1 promotes XBP1 mRNA maturation, enhances chaperonin and CHO transcription, and leads to apoptosis (Acosta-Alvear et al., 2007).

PERK is another protein distributed on the ER membrane. In the early stages of the stress response, PERK inhibits protein translation and synthesis by phosphorylating eukaryotic translation initiation factor 2α (eIF-2α) and increases the production of activating transcription factor 4 (ATF4), which promotes cell survival by reducing protein folding in the ER, inducing the expression of ERS and target genes and participating in redox processes (Harding et al., 2000). However, if ATF4 is active over a period length of time, it may also increase CHOP expression and initiate apoptosis (Han et al., 2013).

ATF6 is a transmembrane protein distributed on the ER membrane. Under nonstress conditions, ATF6 is distributed on the ER membrane in the form of a zymogen. In response to ER stress, ATF6 is transported from the ER to the Golgi apparatus, where it is processed into its active form (Shen et al., 2002). The overexpression of ATF6 activated the transcription of the CHOP and XBP-1 genes as well as that of ER chaperone genes (Yoshida et al., 2000). Most evidence supports the important role of calcium transport from the ER to mitochondria in regulating cellular bioenergy, the production of reactive oxygen species, and the induction of autophagy and apoptosis (Kaufman and Malhotra, 2014). Under ER stress, the Ca^2+^ balance is disrupted and large amounts of Ca^2+^ enter cells and mitochondria. Ca^2+^ influx affects mitochondrial and Bcl-2 family protein activity, thereby inducing apoptosis.

In conclusion, inhibiting the ERS signalling pathway is an effective way to alleviate IDD. However, because ERS is a newly discovered apoptotic pathway, few studies have focused on ERS inhibition. Berberine, a quaternary ammonium alkaloid isolated mainly from the TCM rhizome Coptidis, improves oxidative stress-induced apoptosis by regulating ERS and autophagy in human NP cells (Luo et al., 2019). Panax notoginseng saponins inhibit NPC apoptosis by inhibiting ERS in NPCs (Zheng et al., 2022).

## 3 Cellular pathways

### 3.1 PI3K/AKT pathway

The PI3K-AKT (PKB) signalling pathway is a classical signalling pathway in cells. It is a signalling pathway related to phosphatidylinositol and is derived from RTK mediation. It plays an important role in regulating cell survival. PI3K can produce a variety of membrane-bound PI-3-P molecules after activation. PKB can use its PH domain to bind to these anchor sites and then complete downstream signal transduction. Activated PKB can directly phosphorylate precursor apoptotic proteins such as Bad and has a short-term effect on preventing the activation of apoptotic pathways that lead to cell death. Activated PKB can also have long-term effects, that is, by phosphorylating multiple Ser/Thr residues of the FOXO transcription factor family member FOXO3A, it binds to the cytoplasmic phosphoserine binding protein 14-3-3 and is retained in the cytoplasm, so that it cannot enter the nucleus to cause apoptosis gene transcription, thereby reducing the effect of apoptosis and promoting cell survival (Morovicz et al., 2022).

By activating the PI3K/AKT/mTOR pathway, moracin M might inhibit LPS-induced PI3K and Akt phosphorylation, which leads to the promotion of autophagy and the inhibition of inflammatory mediator production in NPCs (Guo et al., 2018b). Naringin may reduce apoptosis induced damage and mitochondrial dysfunction by increasing PI3K/AKT signalling, since it increases mitochondrial ROS production, reduces matrix metalloproteinase (MMP), and decreases intracellular ATP and mitochondrial ultrastructure changes (Nan et al., 2020). Resveratrol has also been shown to enhance matrix biosynthesis in nucleus pulposus cells through the PI3K/AKT signalling pathway (Jiang et al., 2019; Gao et al., 2018; Wang et al., 2018). Panax notoginseng saponin can protect NPCs against apoptosis via the AKT pathway and autophagy inhibition and ameliorate disc degeneration *in vivo*, indicating its potential as a therapeutic agent for IDD (Guo et al., 2024). Achyranthoside D prevents apoptosis by downregulating caspase-3 and Bax levels while upregulating Bcl-2 expression. This action attenuates the PI3K/AKT/mTOR signalling pathway, leading to increased phosphorylation of Akt and mTOR (Zhang et al., 2022).

### 3.2 MAPK pathway

Mitogen-activated protein kinase (MAPK) is an important transmitter of signals from the cell surface to the interior of the nucleus. The MAPK signalling pathway regulates a variety of important cellular physiological/pathological processes such as cell growth, differentiation, adaptation to environmental stress, and the inflammatory response. MAPKs can be divided into four subfamilies: p38, JNK, ERK and ERK5 (Plotnikov et al., 2011). The MAPK pathway orchestrates apoptosis through dynamic interplay of its ERK, JNK, and p38 subfamilies. ERK predominantly suppresses apoptosis by phosphorylating anti-apoptotic proteins (e.g., Bcl-2, Mcl-1) and activating CREB-mediated survival signals (Wada and Penninger, 2004). Conversely, JNK/p38 activation promotes apoptosis via mitochondrial permeabilization (Bax activation, Cyt c release) and transcriptional induction of pro-apoptotic factors (Bim, FasL) (Tournier et al., 2000). Critically, the apoptotic outcome depends on signal duration, cross-talk with PI3K/AKT pathways, and cellular stress status (Murphy et al., 2002; Franke et al., 2003; Dhanasekaran and Reddy, 2008).

Through the inhibition of p38 MAPK, Bushen Huoxue decoction inhibits the histomorphologic degeneration of the rat lumbar IVD and delays the IDD to a certain extent (Jie et al., 2017). Duhuo Jisheng decoction can reduce the phosphorylation level of P38 MAPK in AF cells, inhibit the production of inflammatory factors, and slow the process of chondrocyte apoptosis mediated by the P38 MAPK signal transduction pathwa to achieve the goal of treating lumbar discogenic LBP (Jiangtao et al., 2012). Duhuo jisheng decoction prevents apoptosis by regulating autophagy and blocking p38 MAPK signalling (Liu et al., 2020). The Liuwei Dihuang pill may protect NP cells, slow the apoptosis of NPCs, and stabilize the cells by inhibiting the effects of the JNK and p38 MAPK pathways, thus it has obvious preventive and treapeutic effects on IDD (Xianbo and Wuji, 2017). The downregulating of p-p38 and NF-κB is one of the mechanisms through which Shentong Zhuyu decoction suppresses apoptosis (Sha et al., 2015). The antiapoptotic effects of baicalein include the upregulation of aggrecan and collagen II; the suppression of NO, PGE2, TNF-α, IL-6, COX-2, iNOS, MMP13 and ADAMTS5; and the inhibition of the NF-κB and MAPK pathways (Jin et al., 2019). Glycyrrhizin prevents IDD through its antiapoptotic and anti-inflammatory effects via the inhibition of HMGB1 via the p38/p-JNK signalling pathway (Liu et al., 2019). Resveratrol inhibits NPC apoptosis in a dose-dependent manner by inhibiting activation of the ERK1/2 signalling pathway, resulting in reduced Bax and caspase-3/cleaved caspase-3, significantly increased Bcl-2, and significantly decreased Bax/Bcl-2 ratios (Zhang et al., 2018b). Sesamin suppresses the activation of the MAPK pathway by inhibiting the phosphorylation of JNK to reduce the expression of catabolic enzymes (MMP-1, MMP-3, MMP-13, ADAMTS-4, and ADAMTS-5) and inflammatory factors (IL-1β, TNF-α, iNOS, NO, COX-2, and PGE2) to protect against apoptosis (Li et al., 2016). Wogonin prevents apoptosis by blocking the Nrf2/ARE and MAPK signalling pathways via the downregulation of inflammatory mediators (iNOS, IL-6 and COX2) and matrix-degrading proteinases (MMP1, MMP3, MMP13 and ADAMTS4) and the upregulation of the ECM (such as collagen II) (Fang et al., 2018a). Moreover, demethoxycurcumin, kaempferol, Rhizoma drynariae total flavonoids, eupatilin, pilose antler peptide and carthamin yellow regulate MAPK pathways to reduce NP cell apoptosis (Lu et al., 2022; Wang et al., 2023b; Zhao et al., 2021; Yang et al., 2022; Dong et al., 2018; Chen et al., 2017). Sodium tanshinone IIA sulfonate and ginsenoside Rg3 inhibit p38 MAPK and reduce NP cell apoptosis (Dai et al., 2021a; Chen et al., 2024).

### 3.3 JAK/STAT pathway

JAK/STAT pathway, a class of intracellular molecules Janus Kinase (JAK), after receiving signals from upstream receptor molecules, is rapidly recruited to the receptor and activated, and activated JAK catalyzes tyrosine phosphorylation of the receptor. The phosphorylated tyrosine on the receptor molecule is the recognition and binding site of signal transducer and activator of transcription (STAT) SH2. STAT also undergoes tyrosine phosphorylation after binding to the receptor. Tyrosine-phosphorylated STAT forms a dimer and enters the nucleus. Interferon binds and activates its receptor to regulate the JAK-STAT pathway. The JAK/STAT pathway exerts dual regulatory roles in apoptosis through STAT isoform-specific signaling (Levy and Darnell, 2002). STAT1 activation promotes apoptosis via transcriptional upregulation of pro-apoptotic factors (e.g., Fas, caspases, IRF1), whereas STAT3/5 enhance survival by inducing anti-apoptotic genes (Bcl-2, Mcl-1, Survivin) (Cao et al., 2015; Wu et al., 2019). This dichotomy is context-dependent, influenced by cytokine milieu (e.g., IFN-γ vs. IL-6), cross-talk with PI3K/mTOR pathways, and epigenetic modulation of SOCS expression. Dysregulated JAK/STAT signaling contributes to apoptosis resistance in IDD and autoimmune disorders.

Duhuo Jisheng decoction inhibits apoptosis in NPC via the JAK/STAT signalling pathway (Kang, 2024). Astragaloside IV (AS IV) and tanshinone (TS IIA) are natural herbal products derived from Astragalus and Salvia miltiorrhiz. It has been shown to reduce NP cell apoptosis through the JAK2/STAT1 pathway by downregulating miR-223 and inhibiting the expression of JAK2 and STAT1 (Du et al., 2021). Salvianolic acid B protects IVDs from oxidative stress-induced IDD by increasing proliferation and attenuating apoptosis via activation of the JAK2/STAT3 signalling pathway (Dai et al., 2021b).

### 3.4 AMPK pathway

The AMP-activated protein kinase (AMPK) signalling pathway is an intracellular signalling pathway that participates in the regulation of cellular metabolism and is closely related to cellular energy metabolism. AMPK is an important protein kinase that is activated by protein kinase-activated protein kinase (LKB1), which regulates cell metabolism and growth in response to the energy state and metabolic state of cells. The activation of AMPK may have important potential therapeutic effects in the treatment of a variety of diseases. The AMPK pathway exerts context-dependent control over apoptosis through metabolic stress sensing (Herzig and Shaw, 2018). Under energy deprivation, AMPK activation triggers pro-apoptotic signaling via p53-Bax axis activation and mTORC1 inhibition (reducing anti-apoptotic Mcl-1 synthesis) (Jones et al., 2005). Conversely, AMPK suppresses apoptosis during oxidative stress by phosphorylating ULK1 to enhance mitophagy (clearance of damaged mitochondria) and inhibiting ACC-mediated lipid peroxidation (Tian et al., 2015). This dual regulation integrates cellular energy status, redox balance, and organelle integrity to determine apoptotic commitment.

Bushen Huoxue Formula regulates autophagy and enhances autophagic flux to suppress excessive ROS production and restore mitochondrial function in an AMPK/SIRT1-dependent manner (Gao et al., 2022). The Yiqi Huoxue Recipe delays IDD by enhancing autophagy and reducing apoptosis and inflammation through the AMPK pathway (Dai et al., 2021c). Apigenin alleviates IDD by enhancing autophagy and reducing apoptosis through the AMPK/mTOR pathway (Xie et al., 2021).

### 3.5 Wnt pathway

The Wnt signalling pathway is a complex network of protein actions whose function is most commonly observed in embryonic development and cancer, but it is also involved in normal physiological processes in adult animals (Lie et al., 2005). The Wnt signalling pathway is a set of signal transduction pathways with multiple downstream channels activated by the binding of the ligand protein Wnt and membrane protein receptors. In this way, the extracellular signal is transmitted to the cell through the activation of the intracellular segment of the cell surface receptor. The Wnt signalling pathway consists of three branches: the canonical Wnt/β-catenin pathway, which activates the expression of target genes in the nucleus; the planar cell polarity pathway, which is involved in JNK activation and cytoskeleton rearrangement; and the Wnt/Ca^2+^ pathway, which activates phospholipase C and protein kinase C. Wnt signaling bidirectionally regulates apoptosis: β-catenin-dependent signaling inhibits apoptosis via Survivin/Bcl-2 activation, while Wnt/Ca^2+^-JNK axis promotes mitochondrial apoptosis. Cellular outcomes depend on Wnt ligand-receptor specificity and coexisting stress signals (Nusse and Clevers, 2017).

Bushen Huoxue decoction can effectively inhibit the protein expression of β-catenin, a key protein in the classical Wnt/β-catenin signalling pathway, to delay the progression of IDD (Fang et al., 2018b). Bushen Huoxue decoction alleviates IDD by regulating NPC proliferation and ECM remodelling through the Wnt signalling pathway (Yang et al., 2019a). Duhuo Jisheng decoction can delay the degeneration of IVD chondrocytes by regulating the Wnt/β-catenin signalling pathway and downregulating the expression of Wnt4, GSK-3β and β-catenin mRNAs and proteins in the degenerative articular cartilage of the rat IVD (Boling et al., 2018). Ginsenoside Rg1 can promote ECM synthesis in degenerative NPCs, inhibit their apoptosis, and improve the IVD via inhibition the Wnt/β-catenin pathway (Yu et al., 2020).

### 3.6 Notch pathway

Notch signalling affects multiple processes in normal cell morphogenesis, including multipotent progenitor cell differentiation, cell apoptosis, cell proliferation, and cell boundary formation. The Notch signaling pathway demonstrates compartment-specific regulatory effects on apoptosis during IDD, particularly within NP cells. Proteolytic cleavage of Notch receptors by γ-secretase generates the Notch intracellular domain (NICD), which orchestrates ECM metabolism and cellular survival through transcriptional regulation (Fortini, 2009). In early IDD stages, Notch activation preserves NP cell viability by upregulating anti-apoptotic Bcl-2 and suppressing caspase-3 activation, potentially through Hes1-mediated stabilization of hypoxia-inducible factors (Liu et al., 2017). Conversely, chronic Notch hyperactivation in degenerative discs correlates with accelerated apoptosis via JAG1-dependent upregulation of pro-death regulators (Bax/p53) and MMP-13-mediated ECM catabolism (Risbud and Shapiro, 2014). This paradoxical behavior appears mechanistically linked to inflammatory microenvironmental cues–TNF-α synergizes with Notch to amplify NF-κB-driven apoptotic signaling while impairing aggrecan synthesis. The pathway’s crosstalk with HIF-1α and TGF-β/Smad3 signaling creates a mechanosensitive regulatory network that modulates NP cell fate under varying mechanical loads. Emerging evidence positions Notch as a potential therapeutic node for IDD, with preclinical models showing that targeted inhibition preserves disc hydration and mitigates apoptosis-associated matrix remodeling. Yougui Wan can inhibit the inflammatory response in IDD rats, reduce the degradation of the ECM, reduce apoptosis in NPCs, and alleviate IDD by regulating the Notch signalling pathway (Ma et al., 2024).

### 3.7 Nrf2 pathway

Nuclear respiratory factor (Nrf)-2 is a transcription factor critical for oxidative stress that binds to antioxidant response elements (AREs) located in the promoter regions of many cytoprotective genes. The Nrf2 pathway predominantly suppresses apoptosis under oxidative stress by transactivating cytoprotective genes (e.g., HO-1, NQO1) that neutralize ROS and inhibit mitochondrial cytochrome c release. This anti-apoptotic effect is mediated through Keap1-Nrf2-ARE axis stabilization of Bcl-2 and suppression of caspase-3/9 activation. Conversely, sustained Nrf2 hyperactivation may paradoxically promote apoptosis via metabolic overload (e.g., NADPH depletion) or crosstalk with p53-mediated pro-death signals. The apoptotic outcome is context-dependent, dictated by stress intensity, cell type-specific redox homeostasis, and tumorigenic status (Taguchi and Yamamoto, 2017).

By modulating the Nrf2 signalling pathway, icariin upregulates the protein expression of Nrf1 and mitochondrial transcription factor, promoting mitochondrial biogenesis in human NPCs and thereby ameliorating IDD (Hua et al., 2020). Myricetin ameliorates IDD by downregulating MMP-13 and ADAMTS and upregulating aggrecan and collagen II through the Nrf2/HO-1/NF-κB signalling pathway (Mao and Fan, 2024). Hyperoside ameliorated TNF-α-induced inflammation, ECM degradation, and ERS-mediated apoptosis by regulating the SIRT1/NF-κB and Nrf2/ARE pathways (Xie et al., 2022).

### 3.8 Other pathways

**FIGURE 2:**
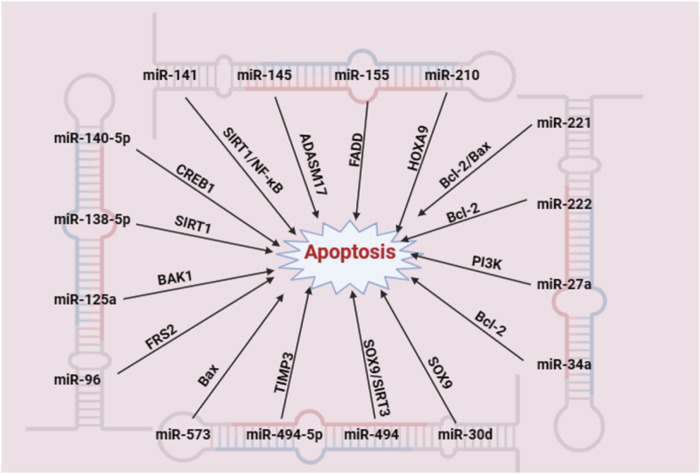
miRNAs participate in IDD by promoting the apoptosis of NPCs (created with BioRender.com). A variety of miRNAs can affect the apoptosis of NPCs by regulating apoptosis-related targets.

A variety of miRNAs have been confirmed to be involved in IDD through NPC apoptosis (Wang et al., 2022). Figure 2 shows the specific process by which miRNAs are involved in NPC apoptosis.

Duhuo Jisheng decoction suppresses apoptosis and mitochondrial dysfunction in human NPCs via the miR-494/SIRT3/mitophagy signalling axis (Liu et al., 2023). Through the modulation of miR-221, lumbar granules were shown to be effective in preventing IDD (Shenghua et al., 2021). Aucubin inhibits ECM degradation in NPC through blocking the miR-140-5p/CREB1 axis (Yang et al., 2019b).

The TLR/MyD88/NF-κB signalling pathway is an important pathway in the inflammatory system of the body; this pathway is widely present in various tissues and cells and participates in the occurrence and regulation of a variety of diseases. Andrographolide (Zhang et al., 2018c) and sparstolonin B (Ge et al., 2018) mitigate IDD through the TLR4/MyD88/NF-κB signalling pathway.

In addition, Duhuo Jisheng decoction (Liu et al., 2018), Yishen Huoxue Tongluo decoction (Xiao et al., 2021), berberine (Lu et al., 2019), curcumin (Cherif et al., 2019), and halofuginone (Luo et al., 2018) can inhibit the apoptosis of NPCs through the Nf-κb signalling pathway to alleviate IDD.

## 4 Discussion and conclusion

Although apoptosis is the earliest identified form of cell death pathway, few comprehensive reviews have focused on the therapeutic potential of TCM in alleviating IDD through antiapoptotic mechanisms. This review summarizes recent advances in TCM-based strategies for preventing apoptosis in IDD. We explore how TCM compounds or their active ingredients exert protective effects through three major apoptotic pathways: the exogenous death receptor pathway, the endogenous mitochondrial pathway, and the ERS pathway. The endogenous apoptotic pathway, primarily regulated by mitochondria and the ER, along with the exogenous apoptotic pathway, which is mediated by death receptors, are both discussed in detail. In addition, key apoptosis-related signalling pathways, such as the PI3K/AKT, MAPK, JAK/STAT, and AMPK pathways, are also explored. Collectively, these findings indicate that TCM exert their antiapoptotic effects through diverse molecular mechanisms.

This review underscores the therapeutic potential of TCM in mitigating IDD through apoptosis modulation, providing a mechanistic framework for future investigations. However, critical gaps persist in understanding the multi-targeted synergy of TCM across interconnected signaling pathways (e.g., Wnt/β-catenin-NF-κB crosstalk) and its temporal regulation of apoptotic-anoikis networks. Notably, the pathogenesis of IDD is highly complex, with apoptosis being only one aspect. While multiple PCD pathways in IVD cells are well-characterized, single-pathway inhibition often fails to prevent cell demise, suggesting a need for multi-targeted therapeutic approaches. In recent years, various cell death modes beyond apoptosis—including ferroptosis, pyroptosis and cuproptosis—have been implicated in IDD pathogenesis. However, there is still a lack of research on the correlation of multiple signalling pathways, such as the synergistic effect of TCM on different signalling pathways and whether apoptosis and other types of programmed cell death are regulated at the same time and the exact mechanism involved is a largely uncharted territory. To advance global acceptance of TCM-based IDD therapies, rigorous mapping of its cell death regulatory atlas through multi-omics integration (scRNA-seq, spatial metabolomics) and AI-driven network pharmacology is imperative. While this review has focused on pharmacological TCM interventions targeting apoptotic pathways, evidence suggests that non-pharmacological modalities like scraping therapy mitigate IDD through distinct biomechanical and immunomodulatory mechanisms. We therefore commit to future exploration of multimodal therapeutic integration—combining herbal compounds with physical interventions—to exploit complementary anti-IDD effects. Moreover, future clinical trials are essential to validate the efficacy and safety of TCM-based therapies in IDD patients, particularly those targeting multiple apoptotic pathways.Only by resolving these multidimensional mechanisms can TCM formulations achieve clinical translatability as precision modulators of disc homeostasis. As the field of antiapoptotic research progresses, there is increasing urgency to identify safe, effective, and economically viable compounds from the vast array of natural resources, including plants and animals, to support the prevention and management of IDD. This approach not only aligns with the principles of sustainable development but also holds the promise of enriching the therapeutic armamentarium against IDD.

## 5 Limitations

This study has some limitations. First, our literature screening was restricted to three databases (PubMed, CNKI, and Web of Science), potentially omitting relevant studies from regional repositories or unpublished datasets. Second, the heterogeneity of experimental designs across included preclinical studies (e.g., varying TCM dosage regimens, animal models of IDD) complicate direct comparisons and meta-analyses. Third, while we systematically summarized TCM-mediated anti-apoptotic mechanisms, most evidence derives from cell/animal models, and clinical trials validating these findings remain scarce. Fourth, the multifactorial nature of TCM compounds (e.g., multi-target effects, bioavailability variations) was not fully addressed in mechanistic analyses, highlighting a need for standardized protocols to isolate active ingredients. Finally, publication bias may exist, as negative results or failed interventions are less frequently reported. Despite these limitations, this review provides the first comprehensive synthesis of TCM’s anti-apoptotic roles in IDD pathogenesis, integrating molecular targets across death receptor, mitochondrial, and ERS pathways. Our cross-database approach ensured coverage of both western and Chinese scholarly outputs, while mechanistic categorization offers actionable insights for future drug development. The emphasis on natural compounds aligns with global trends toward sustainable therapeutics.
